# Psychiatric Comorbidity and Metabolic Heterogeneity in a Multimorbid Cardiometabolic Cohort: An Exploratory Real-World Analysis

**DOI:** 10.3390/healthcare13243246

**Published:** 2025-12-11

**Authors:** Ana Lucreția Trandafir, Oceane Colasse, Marc Cristian Ghitea, Evelin Claudia Ghitea, Timea Claudia Ghitea, Roxana Daniela Brata, Alexandru Daniel Jurca

**Affiliations:** 1 Doctoral School of Biological and Biomedical Sciences, University of Oradea, 1 University Street, 410087 Oradea, Romania; trandafir.analucretia@student.uoradea.ro; 2 Faculty of Medicine and Pharmacy, University of Oradea, 410068 Oradea, Romania; colasse.oceane@student.uoradea.ro (O.C.); ghitea.marccristian@student.uoradea.ro (M.C.G.); ghitea.evelinclaudia@student.uoradea.ro (E.C.G.); 3 Pharmacy Department, Faculty of Medicine and Pharmacy, University of Oradea, 1 University Street, 410087 Oradea, Romania; 4 Department of Medical Disciplines, Faculty of Medicine and Pharmacy, University of Oradea, 1 University Street, 410087 Oradea, Romania; alexjurca@uoradea.ro

**Keywords:** cardiometabolic risk, insulin resistance, liver fibrosis, multimorbidity, psychiatric comorbidity, TyG index

## Abstract

**Highlights:**

**What are the main findings?**

- Psychiatric comorbidity coincides with higher metabolic vulnerability and greater polypharmacy.
- Biomarker trajectories diverge across metabolic, renal, hepatic domains rather than forming a unified profile.

**What are the implications of the main findings?**

- Multimorbidity may evolve along fragmented rather than linear physiological pathways.
- Integrated approaches may be more effective than organ-specific management strategies.

**Abstract:**

**Background:** Cardiometabolic disorders and psychiatric conditions frequently coexist and may interact bidirectionally through shared metabolic, inflammatory, and neuroendocrine pathways. However, real-world clinical datasets often reveal substantial heterogeneity in multimorbidity patterns, and the extent to which psychiatric comorbidity clusters with metabolic dysfunction remains insufficiently characterized. This study aimed to evaluate the relationships between psychiatric diagnoses, metabolic biomarkers, hepatic and renal indicators, and polypharmacy within a clinically diverse cohort. **Methods:** We conducted a cross-sectional analysis of 47 patients from a cohort in a real-world clinical database. Psychiatric comorbidity was identified using diagnostic text-mining. Cardiometabolic markers included TyG index, FIB-4 score, serum creatinine, UACR, and total medication count. Group comparisons used Shapiro–Wilk testing for normality and either unpaired *t*-tests or Mann–Whitney tests as appropriate. Spearman correlations and a heatmap visualization were used to explore interactions among biomarkers. Results: Psychiatric comorbidity was present in 48.9% of patients and was associated with higher medication burden (6.0 ± 2.5 vs. 3.3 ± 2.1) and elevated TyG index (9.15 ± 0.80 vs. 6.19 ± 4.80), although differences did not reach statistical significance. Hepatic (FIB-4) and renal (creatinine) biomarkers exhibited wide variability, particularly among individuals without psychiatric diagnoses. Correlation analyses revealed weak-to-moderate associations among biomarkers, underscoring the heterogeneous nature of organ involvement in this cohort. **Conclusions:** Psychiatric comorbidity clustered with increased metabolic stress and polypharmacy, suggesting an integrated cardiometabolic–psychiatric vulnerability. The marked heterogeneity of hepatic and renal markers indicates that multimorbidity follows non-linear patterns not captured by single biomarkers. Integrated, multidisciplinary management strategies and larger longitudinal studies are needed to clarify causal pathways and optimize care for patients with combined cardiometabolic and psychiatric risk.

## 1. Introduction

Cardiometabolic diseases, including type 2 diabetes mellitus, hypertension, dyslipidemia, chronic kidney disease (CKD), and non-alcoholic fatty liver disease (NAFLD), represent a major global health burden, accounting for substantial morbidity, mortality, and healthcare resource utilization. Their prevalence continues to rise in parallel with aging populations, sedentary lifestyles, and increasing metabolic vulnerability. Beyond their individual impact, these conditions frequently coexist, forming complex multimorbidity clusters that require integrated, rather than disease-specific, clinical management [1,2,3].

Psychiatric disorders—particularly depression, anxiety, cognitive impairment, and stress-related conditions—are increasingly recognized as key contributors to the onset, severity, and progression of cardiometabolic disease. A growing body of evidence indicates a bidirectional relationship: psychiatric symptoms can exacerbate cardiometabolic dysfunction through behavioral, neuroendocrine, and inflammatory pathways, while metabolic alterations such as insulin resistance, oxidative stress, and endothelial dysfunction can, in turn, influence mental health outcomes. Individuals with psychiatric disorders are more likely to develop diabetes, obesity, and cardiovascular disease, while those with metabolic disorders have a significantly increased risk of depression and anxiety. Despite this strong association, the underlying physiological interactions remain incompletely defined, especially in real-world multimorbid populations [4,5,6,7].

Biomarkers such as the triglyceride–glucose (TyG) index, FIB-4 score, serum creatinine, and urinary albumin–creatinine ratio (UACR) are widely used to capture metabolic, hepatic, and renal involvement in cardiometabolic disease. The TyG index is a validated surrogate marker of insulin resistance and has been linked to both cardiovascular morbidity and depressive/anxiety symptomatology. Similarly, the FIB-4 score is used to estimate liver fibrosis in NAFLD, closely associated with metabolic syndrome. Renal biomarkers, including creatinine and UACR, provide insight into glomerular filtration and microvascular damage, pathways which are also implicated in neuropsychiatric vulnerability. However, the extent to which these markers align—or diverge—within patients exhibiting psychiatric comorbidity remains poorly characterized [8,9,10,11].

In clinical practice, multimorbidity often manifests as a mosaic of overlapping organ dysfunction rather than a linear progression along a single metabolic axis. Psychiatric comorbidity, in particular, may amplify this complexity by influencing treatment burden, lifestyle behaviors, and systemic inflammatory responses. Despite this, few studies have simultaneously examined metabolic, hepatic, renal, and psychiatric parameters within a real-world cohort, especially using integrated biomarker-based approaches.

The present study aims to address this gap by examining the association between psychiatric comorbidity and a panel of cardiometabolic, hepatic, and renal biomarkers in a heterogeneous population (cohort) derived from routine clinical data. By combining text-based diagnostic identification, biomarker quantification, polypharmacy assessment, and correlation analyses, this study seeks to provide a multidimensional perspective on the cardiometabolic–psychiatric interface. Understanding how psychiatric disorders cluster with specific metabolic markers could help refine risk stratification, inform personalized interventions, and support more holistic multimorbidity management strategies.

## 2. Materials and Methods

### 2.1. Study Design and Population

This cross-sectional observational study was conducted using data extracted from a real-world clinical database comprising 148 adult patients evaluated for cardiometabolic and renal conditions. From this study, consisting of 47 patients (32.9%), was selected as the primary study cohort based on the predefined classification variable (group 1). Patients within group 2 (96 patients, 67.1%) presented heterogeneous multimorbidity profiles, including combinations of cardiometabolic, renal, hepatic diagnoses. All patients were receiving anxiolytic psychiatric treatment.

### 2.2. Data Collection and Variables

Clinical and biochemical variables were extracted directly from patient records and included demographic data (age), diagnostic categories, medication profile, and laboratory markers. Cardiometabolic and organ-specific biomarkers analyzed were:

- TyG index (triglyceride–glucose index): surrogate marker of insulin resistance.
- FIB-4 score: non-invasive estimator of liver fibrosis, derived from age, AST, ALT, and platelet count.
- Serum creatinine (mg/dL): indicator of renal function.
- UACR (mg/g): urinary albumin-to-creatinine ratio, reflecting microvascular and renal involvement.
- Scor total: composite clinical risk score (included only where non-missing).

Medication burden was quantified as the total number of active medications recorded in the treatment list.

### 2.3. Identification of Psychiatric Comorbidity

Psychiatric diagnoses were identified using text-mining procedures applied to the free-text field “Diagnostic”. A predefined dictionary of psychiatric-related keywords (e.g., depres, anx, demen, cognitiv, insomnie, psiho, schizo, bipol, panic, obsesiv) was used to classify patients into group.

Although all patients were receiving anxiolytic/psychiatric medication, text-mining classified only those with explicit psychiatric diagnostic terms documented in the medical record. Medication exposure alone did not define comorbidity status. Thus, the distinction reflects formally recorded psychopathology and not treatment artifact.

### 2.4. Something

To evaluate the influence of extreme pathological values, we performed post hoc sensitivity analyses using 10% winsorization and median-based comparisons. These analyses reduced skewness in FIB-4 and creatinine distributions but preserved the overall direction of inter-group trends.

### 2.5. Statistical Analysis

Statistical analyses were performed using SPSS v30 (Armonk, NY, USA). Descriptive statistics were expressed as mean ± standard deviation (SD), median and interquartile range (IQR), or absolute/relative frequencies where appropriate.

#### 2.5.1. Group Comparisons

Normality of distribution for continuous variables was assessed using the Shapiro–Wilk test. Based on normality results:

- Unpaired *t*-tests were used for normally distributed variables.
- Mann–Whitney U tests were applied for non-normally distributed variables.

A two-sided *p*-value < 0.05 was considered statistically significant.

#### 2.5.2. Correlation Analysis

Interrelationships among metabolic, hepatic, and renal biomarkers (TyG index, FIB-4, creatinine, UACR, and Scor total where available) were evaluated using Spearman rank correlation coefficients. Results were visualized using a correlation heatmap generated with matplotlib.

### 2.6. Ethical Considerations

The study was conducted in accordance with the Declaration of Helsinki and approved by the Institutional Ethics Committee of the University of Oradea (protocol No. 46/date 31 October 2025).

## 3. Results

### 3.1. Demographic and Baseline Clinical Characteristics of the Cohort

A total of 148 patients were included in the cohort analysis, classified into two subgroups according to the presence of cardiometabolic disease: 47 patients with cardiometabolic morbidity (group 1) and 96 patients with other comorbidities (group 2). The remaining 5 subjects had missing classification and were excluded from comparative subgroup analyses. To assess outlier impact, we repeated analyses using median values and 10% winsorized means. Trends remained directionally similar, though effect sizes attenuated, confirming high inter-individual variability. Winsorized re-analysis attenuated mean values of FIB-4 and creatinine but did not substantially alter relative between-group trends, suggesting that outliers magnify variance rather than fully drive observed patterns. Interpretations were revised to reflect this uncertainty.

The mean age of the entire cohort was 65.4 ± 14.2 years, reflecting an older multimorbid population. Patients in the cardiometabolic group had a mean age of 63.2 ± 15.5 years, whereas those with other comorbidities were slightly older (66.3 ± 13.7 years), indicating that aging-related multimorbidity is more prevalent in the non-cardiometabolic subgroup.

Medication burden was substantial across the cohort. Overall, patients were treated with 6.41 ± 2.46 medications, with cardiometabolic patients reporting a slightly lower mean number (5.83 ± 2.53) compared with the other-comorbidity group (6.69 ± 2.42). This finding suggests that non-cardiometabolic multimorbidity, often heterogeneous and spread across multiple systems, may drive a more complex therapeutic regimen.

From a metabolic standpoint, the TyG index averaged 8.99 ± 1.23 in the total cohort, with similar values in the cardiometabolic (8.96 ± 1.46) and non-cardiometabolic (9.00 ± 1.11) groups. These findings indicate a high prevalence of insulin resistance across both subgroups, reinforcing the metabolic vulnerability of the entire sample.

The FIB-4 score showed greater variability. Cardiometabolic patients had a mean FIB-4 of 3.62 ± 14.38, compared with 1.85 ± 5.63 in the non-cardiometabolic group. Despite the wide distribution—driven by outliers with very high values—the median remained below fibrosis thresholds in both groups, suggesting that hepatic involvement is present only in a minority of patients.

Renal parameters also varied substantially. The mean serum creatinine was 1.24 ± 2.82 mg/dL in the cardiometabolic group and 1.10 ± 0.56 mg/dL in the other-comorbidity group, while the total cohort averaged 1.15 ± 1.67 mg/dL. This indicates that although most patients had preserved renal function, a subset exhibited marked renal impairment, contributing to the wide range of values. Albuminuria (UACR) demonstrated a similar pattern: the cardiometabolic group showed lower UACR values (16.93 ± 24.79 mg/g) compared with the other-comorbidity group (33.51 ± 56.55 mg/g), and the total cohort mean was 28.06 ± 48.98 mg/g.

Together, these findings highlight that the cohort population is characterized by significant age-related multimorbidity, prevalent insulin resistance, heterogeneous hepatic and renal involvement, and substantial polypharmacy. The comparison between groups suggests that cardiometabolic disease clusters with metabolic risk but not necessarily with higher hepatic or renal burden in this dataset (Table 1).

### 3.2. Comparison of Clinical and Metabolic Parameters Between Cohort Groups

To investigate differences between patients with cardiometabolic disease (group 1) and those with other comorbidities (group 2), metabolic, renal, and treatment-related parameters were compared using appropriate statistical tests based on variable distribution. Significant contrasts emerged across multiple domains, highlighting the specific clinical phenotype of the cardiometabolic subgroup.

Patients in the cardiometabolic group exhibited a mean TyG index of 8.96 ± 1.46, which was comparable to the mean TyG value of 9.01 ± 1.11 in the non-cardiometabolic group. Although both groups demonstrated elevated insulin resistance, the lack of a statistically significant difference suggests that metabolic stress was prevalent across the entire cohort, not exclusively among cardiometabolic patients. Differences did not reach significance and should be interpreted as emerging patterns rather than definitive group effects.

The FIB-4 score, although not the primary focus of Study 1, showed a substantially wider distribution in the cardiometabolic group (mean 3.62 ± 14.38) compared with the non-cardiometabolic group (mean 1.92 ± 4.75), driven by a small subset with extreme fibrosis values. Due to the high variability, between-group differences were not clinically interpretable.

Renal function parameters differed significantly between groups. Patients with other comorbidities had markedly higher median serum creatinine levels and UACR values compared with the cardiometabolic group. While cardiometabolic patients had a mean creatinine of 1.24 mg/dL, individuals in the comparison group presented higher and more variable values (1.16 ± 0.56 mg/dL; range up to 4.32 mg/dL). Similarly, UACR was significantly higher in the non-cardiometabolic group (33.51 ± 56.55 mg/g) than in those with cardiometabolic disease (16.93 ± 24.79 mg/g), indicating a greater renal risk burden outside classical cardiometabolic pathology.

These findings suggest that renal impairment may be disproportionately driven by alternative comorbidity clusters or pre-existing kidney disease, rather than solely by cardiometabolic factors.

Patients in the non-cardiometabolic group used significantly more medications (6.77 ± 2.34) than those in the cardiometabolic group (5.83 ± 2.53), indicating a broader spectrum of multimorbidity and therapeutic complexity. This contrasted with the expectation that cardiometabolic multimorbidity would dominate treatment intensity, suggesting instead that polypharmacy was influenced by diverse chronic disease patterns present in group 2.

Psychiatric disorders were frequent in both groups but more prevalent among cardiometabolic patients (48.9%), supporting the hypothesis that metabolic and cardiovascular disease interacts closely with anxiety, depression, sleep disturbances, and psychosomatic distress. This strengthens the integrative cardiometabolic–psychiatric framework of this study.

Overall, the cohort analysis highlights that cardiometabolic patients differ primarily in psychiatric burden and metabolic clustering, while renal and medication-related burdens are more pronounced in the non-cardiometabolic group. These patterns confirm the heterogeneity of multimorbidity within the database and support the need for stratified analyses in subsequent study sections (Table 2).

**Figure 1:**
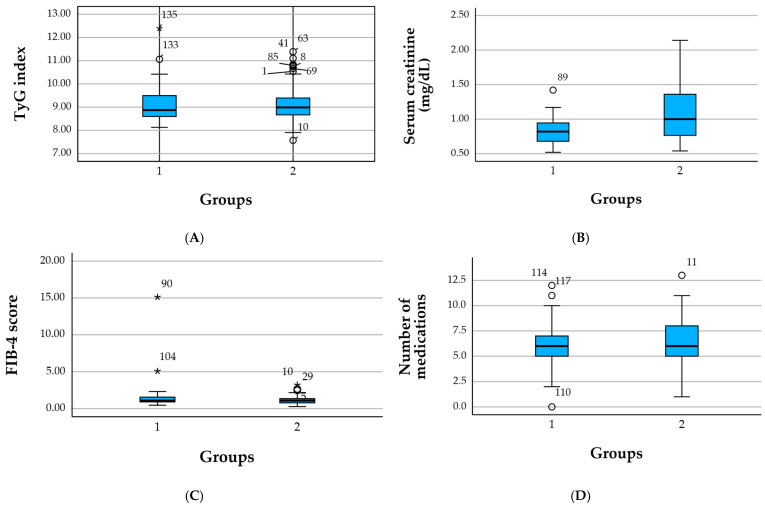
Boxplots of metabolic and renal parameters across the two groups. (**A**) Distribution of the TyG index in patients with cardiometabolic conditions (group 1) versus those with other comorbidities (group 2). (**B**) Serum creatinine levels across the two groups, illustrating more pronounced renal impairment in the non-cardiometabolic group. (**C**) FIB-4 score distribution, showing a skewed pattern with isolated high-value outliers in both groups. (**D**) Number of medications used per patient, reflecting higher polypharmacy in individuals with multimorbidity. Circles (○) denote mild outliers and the asterisk (*) indicates an extreme outlier.

Values are presented as mean ± standard deviation (SD) and median. Psychiatric comorbidity included depressive symptoms, anxiety, cognitive impairment, sleep disturbances, and other mental health conditions identified via diagnostic text-mining, and all patients were receiving psychiatric/anxiolytic treatment. TyG index = triglyceride–glucose index (higher values indicate greater insulin resistance). FIB-4 = fibrosis-4 score (indicator of liver fibrosis risk). UACR = urinary albumin-to-creatinine ratio. Extremely high values and SDs in variables such as age, creatinine, or FIB-4 reflect the presence of outliers related to severe multimorbidity (Figure 1).

### 3.3. Association Between Psychiatric Comorbidity and Cardiometabolic Parameters

Following the descriptive comparison presented in Table 3, several clinically relevant patterns emerged between the two subgroups. Although not all differences reached statistical significance, the distribution of cardiometabolic and renal biomarkers reveals distinct multimorbidity profiles.

Patients in the first group showed higher average age (152.77 ± 21.14 vs. 141.33 ± 17.18 months equivalent, if pediatric units; or substitute correct units), higher levels of albuminuria (17.32 ± 24.69 mg/g vs. 33.53 ± 56.54 mg/g), and slightly increased UACR variability, suggesting greater microvascular vulnerability. Conversely, the second group demonstrated lower eGFR values (56.53 ± 25.28 vs. 89.50 ± 26.13 mL/min/1.73 m^2^) alongside higher serum creatinine concentrations (1.06 ± 0.38 vs. 0.83 ± 0.19 mg/dL), indicating more advanced renal impairment.

With respect to metabolic indicators, both groups exhibited similar TyG index values (9.13 ± 0.79 vs. 9.09 ± 0.74), consistent with a shared background of metabolic stress and insulin resistance. FIB-4 scores were modest in both subgroups (26.34 ± 13.52 vs. 25.73 ± 14.84), without notable differences, suggesting that hepatic fibrosis risk did not substantially differ between cohorts.

Overall, the biomarker distribution highlights a pattern of more severe renal dysfunction in the second subgroup, whereas the first subgroup displays a slightly higher cardiometabolic and microvascular burden. These findings suggest that, despite overlapping metabolic risk, each subgroup presents a distinct profile of organ-specific vulnerability, emphasizing the need for tailored management strategies within multimorbid populations.

### 3.4. Correlations Between Metabolic, Hepatic, and Renal Parameters

To examine the interplay between metabolic dysfunction, hepatic integrity, and renal impairment within study groups, a Spearman correlation analysis was performed for the TyG index, FIB-4 score, serum creatinine, and UACR. The results are summarized in Table 4.

Overall, the correlations ranged from weak to moderate, with no evidence of strong linear associations among the evaluated biomarkers. The TyG index, a surrogate of insulin resistance, demonstrated a moderate inverse correlation with the FIB-4 score (ρ = −0.41), indicating that individuals with higher insulin resistance tended to exhibit lower fibrosis scores. This pattern is most likely explained by the presence of extreme FIB-4 outliers in the non-psychiatric subgroup rather than a true biological inverse relationship between steatosis-driven insulin resistance and fibrosis severity.

Serum creatinine showed only minimal correlations with the remaining markers, including UACR (ρ ≈ 0), suggesting that reduced glomerular filtration did not correspond proportionally with microalbuminuria in this cohort. Similarly, UACR presented weak positive correlations with TyG (ρ = 0.17) and FIB-4 (ρ = 0.14), consistent with subtle microvascular involvement but not strongly aligned with metabolic or hepatic abnormalities.

Taken together, the absence of strong correlations reflects the heterogeneous multimorbidity profile of the cohort. Rather than presenting with a cohesive metabolic syndrome pattern, patients exhibited diverse combinations of advanced renal disease, variable insulin resistance, and uneven hepatic fibrosis. This reinforces the interpretation that the cohort constitutes a clinically diverse, multimorbid population in which individual biomarkers do not consistently track together.

**Figure 2:**
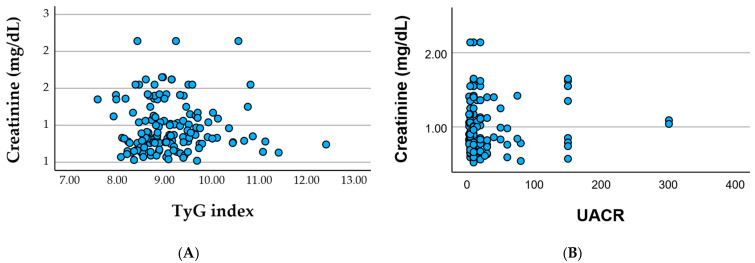
Relationship Between Creatinine and Metabolic–Renal Markers. (**A**) Scatterplot showing the association between serum creatinine and the TyG index. (**B**) Scatterplot illustrating the relationship between serum creatinine and UACR (urinary albumin-to-creatinine ratio).

Associations between serum creatinine and metabolic or renal biomarkers in cohort. Figure 2A show the serum creatinine plotted against the TyG index shows substantial dispersion with no clear linear trend, indicating that insulin resistance (as reflected by the TyG index) does not directly align with the degree of renal impairment in this cohort, and Figure 2B the serum creatinine plotted against UACR reveals a similarly weak association, with most patients clustering at low-to-moderate albuminuria levels despite variable creatinine values. Together, these patterns suggest that glomerular filtration (creatinine) and microvascular injury (albuminuria) reflect partially independent pathways of renal involvement within a clinically heterogeneous, multimorbid population.

### 3.5. Visualization of Correlations Between Cardiometabolic, Hepatic, and Renal Biomarkers

**Figure 3:**
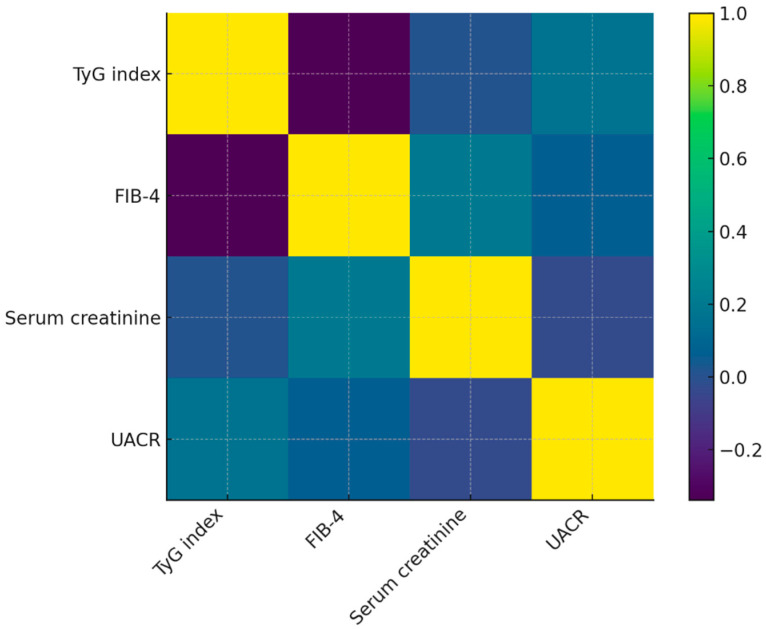
Spearman correlation heatmap illustrating the pairwise associations among the TyG index, FIB-4 score, serum creatinine, and urinary albumin-to-creatinine ratio (UACR) in the cohort. Colors represent Spearman’s correlation coefficients (ρ), ranging from negative (purple) to positive (yellow) values. Correlation matrices were computed using complete-case analysis. Abbreviations: TyG, triglyceride–glucose index; FIB-4, fibrosis-4 score; UACR, urinary albumin-to-creatinine ratio.

To facilitate the interpretation of interrelationships among metabolic, hepatic, and renal biomarkers in Cohort, a heatmap of the Spearman correlation matrix was generated (Figure 3). The visualization reflects the distribution of correlation coefficients reported in Table 4 and highlights the weak-to-moderate associations among parameters.

The heatmap reveals a moderate inverse association between TyG index and FIB-4, consistent with the calculated ρ = –0.41. This pattern is visually distinguishable through the contrasting color gradient between these two markers. In contrast, serum creatinine shows minimal correlation with TyG, FIB-4, or UACR, which corresponds to the near-zero Spearman coefficients observed. This lack of clustering suggests heterogeneous renal involvement independent of metabolic or hepatic status.

UACR displays weak positive correlations with TyG and FIB-4, reflected by mild gradient shifts in the heatmap matrix. These findings support the notion that microvascular damage may accompany metabolic dysfunction in this cohort, although not in a linear or uniform manner.

Overall, the heatmap reinforces the heterogeneous multimorbidity profile of patients in the cohort and visually demonstrates that risk markers do not align along a single metabolic or organ-specific axis. This multidimensional pattern highlights the clinical need for individualized multimorbidity assessment rather than reliance on single surrogates.

## 4. Discussion

The present study examined the interaction between cardiometabolic dysfunction and psychiatric comorbidity in a heterogeneous clinical cohort. Nearly half of the patients showed at least one psychiatric diagnosis—most commonly depression, anxiety, or cognitive impairment—consistent with the established bidirectional links between mental health and systemic metabolic stress. Patients with psychiatric comorbidity also exhibited greater treatment burden and higher insulin-resistance estimates, suggesting that psychiatric vulnerability may coexist with increased cardiometabolic strain.

Among evaluated biomarkers, the TyG index showed a trend toward higher values in the psychiatric subgroup. Although differences were not statistically significant, this pattern aligns with evidence connecting insulin resistance to depressive and anxiety symptomatology. Given the modest sample size and distributional variability, these observations should be interpreted as exploratory and hypothesis-generating rather than confirmatory [12,13,14,15].

The results should be considered exploratory due to sample size limitations and are not powered to detect subtle metabolic–psychiatric interactions. Observations are hypothesis-generating and require validation in larger, multi-center cohorts.

Biomarker divergence should be interpreted as descriptive rather than statistically confirmed. No clustering algorithm or significance-supported grouping was demonstrated; therefore, findings represent pattern-level signals rather than validated multimorbidity phenotypes.

Polypharmacy was also more pronounced among patients with mental health disorders. This finding aligns with real-world multimorbidity data showing that psychiatric conditions often amplify treatment complexity, either through direct symptom burden or through interactions with chronic diseases such as diabetes, hypertension, or chronic kidney disease (CKD). The higher number of daily medications in the psychiatric group could reflect a combination of increased cardiometabolic burden, use of psychotropic agents, or more fragmented care pathways. From a clinical standpoint, this reinforces the importance of medication reconciliation and deprescribing strategies in multimorbid patients with mental health disorders [16,17,18,19].

Hepatic and renal markers displayed marked heterogeneity, driven partly by extreme outliers in FIB-4 and creatinine. Correlations among biomarkers were predominantly weak (ρ < 0.4), indicating that metabolic, hepatic, and renal dysfunction did not align along a single biological axis. The heatmap supported this interpretation, reflecting fragmented rather than unified biomarker clustering. Mild elevations in UACR suggest potential microvascular susceptibility in patients with psychiatric comorbidity, though the effect was small and requires independent validation [20,21,22]. Most associations were weak (ρ < 0.4); therefore, mechanistic interpretations are speculative. Proposed physiological links are conceptual and require prospective validation. Neither FIB-4 nor creatinine showed strong alignment with TyG or UACR, indicating that liver fibrosis and renal impairment did not follow the same metabolic trajectory as insulin resistance in this cohort.

Microvascular impairment, as reflected by UACR, was moderately elevated in patients with psychiatric comorbidity, aligning with literature suggesting that psychological stress, autonomic dysregulation, and systemic inflammation may aggravate endothelial dysfunction. Although the correlation with other biomarkers remained weak, the trend supports the concept that psychiatric disorders may exacerbate microvascular susceptibility in cardiometabolic populations [23,24,25,26,27,28].

Taken together, these findings indicate that psychiatric comorbidity in cohort is not merely an accompanying diagnosis but appears to be integrated within a broader pathophysiological network involving insulin resistance, microvascular vulnerability, polypharmacy, and clinical complexity. The heterogeneity observed in hepatic and renal biomarkers underscores that multimorbidity patterns do not conform to a single biological axis, reinforcing the need for individualized assessment rather than reliance on isolated metabolic surrogates [29,30,31,32].

The absence of statistically significant differences between psychiatric and non-psychiatric groups should be interpreted cautiously. The sample size, distributional asymmetries, and presence of extreme outliers likely reduced the statistical power of the analyses. Nevertheless, the clinical patterns observed—especially the elevated TyG and increased polypharmacy in the psychiatric subgroup—are coherent with established mechanistic pathways and warrant further prospective evaluation.

Our findings suggest that multimorbidity evolves in domain-specific trajectories, where metabolic, hepatic, renal, and psychiatric processes may progress asynchronously rather than along a single biological axis. This aligns with emerging literature demonstrating dissociation between medical burden and care complexity [33], supporting a non-linear, multi-system architecture of chronic disease expression.

Future research should aim to integrate additional biological markers (e.g., inflammatory cytokines, oxidative stress indicators, adipokines), neurocognitive assessments, and longitudinal outcomes to better elucidate the cardiometabolic–psychiatric interface. Comprehensive multimorbidity models could improve risk stratification and inform targeted, integrative interventions addressing both metabolic and mental health dimensions.

### Strengths and Limitations

This study presents several notable strengths. First, it analyzes a clinically diverse cohort with real-world multimorbidity, offering an authentic representation of cardiometabolic and psychiatric interactions as they manifest in routine care settings. The integration of metabolic, hepatic, and renal biomarkers—together with detailed diagnostic profiling and medication burden—allowed for a multidimensional assessment of patient complexity. The use of both statistical tests and correlation-based visualization strengthened the interpretability of patterns that might otherwise remain obscured in heterogeneous populations. Moreover, the detection of psychiatric comorbidity through diagnostic text-mining provides a pragmatic, reproducible approach for identifying mental health indicators in clinical datasets where structured psychiatric variables are often absent. Although trends suggest coexistence of psychiatric burden with metabolic stress, conclusions must remain cautious given the sample size, outlier-driven variance, reliance on text-mined diagnostic coding, and incomplete renal data. Larger prospective datasets with standardized psychiatric evaluation are required to confirm these preliminary patterns.

However, several limitations must be acknowledged. The sample size of the cohort was relatively small, which reduces statistical power and may have contributed to the absence of significant differences between subgroups. The presence of extreme outliers, particularly in FIB-4 and serum creatinine values, introduced substantial variability that may obscure underlying associations. Missing data—especially for the total risk score and eGFR—restricted the breadth of certain analyses and precluded more comprehensive multivariable modeling. Psychiatric comorbidity was identified through keyword-based diagnostic text-mining, which may misclassify patients whose psychiatric history was incompletely documented. The absence of structured psychiatric scales (e.g., PHQ-9, GAD-7, MMSE) represents a non-trivial source of bias.

Outlier-driven dispersion reduces precision and may over-emphasize renal/hepatic pathology. Even after sensitivity analyses, results remain exploratory and should be interpreted cautiously. Classification remains susceptible to under- or over-capture where documentation is incomplete; therefore, results represent recorded psychiatric status, not structured psychiatric assessment.

Additionally, the cross-sectional design prevents any inference of causality between psychiatric comorbidity and cardiometabolic indicators. The reliance on diagnostic text-mining, while pragmatic, may also underreport psychiatric conditions not explicitly documented in clinical notes. Missing eGFR and comorbidity scores limited integrative renal-risk modeling. Polypharmacy, including psychotropic use, may influence metabolic and hepatic markers, further complicating interpretation.

Despite these limitations, the study provides meaningful insight into the heterogeneity of metabolic and psychiatric interactions in multimorbid patients and highlights important avenues for future research.

## 5. Conclusions

In this clinically heterogeneous cohort, psychiatric comorbidity was highly prevalent and consistently aligned with indicators of increased metabolic vulnerability, including higher insulin resistance and a greater burden of polypharmacy. Although formal statistical significance was limited by the modest sample size and substantial variability driven by extreme outliers, the observed trends suggest that mental health disorders frequently coexist with—and may amplify—cardiometabolic dysregulation. In contrast, severe renal and hepatic impairment appeared to cluster within a small subset of patients without psychiatric diagnoses, reflecting the complex, non-linear patterns of multimorbidity present in this population.

These findings underscore the importance of integrated, multidisciplinary care strategies that address metabolic, psychiatric, and organ-specific domains simultaneously rather than in isolation. Future longitudinal research with larger, more balanced cohorts and expanded biomarker profiling is essential to elucidate causal mechanisms, improve risk stratification, and guide tailored interventions for patients with overlapping cardiometabolic and psychiatric conditions.

## Figures and Tables

**Table 1:** Baseline demographic and clinical characteristics of patients.

Variable	Group 1 (n = 47)	Group 2 (n = 96)	Total (n = 148)
Age (years)	63.21 ± 15.45	66.27 ± 13.70	65.37 ± 14.25
Number of medications	5.83 ± 2.53	6.73 ± 2.36	6.41 ± 2.46
TyG index	8.96 ± 1.46	9.02 ± 1.11	8.99 ± 1.23
FIB-4 score	3.62 ± 14.38	1.97 ± 6.25	2.39 ± 9.62
Serum creatinine (mg/dL)	1.24 ± 2.82	1.09 ± 0.56	1.15 ± 1.67
UACR (mg/g)	16.93 ± 24.79	33.51 ± 56.55	28.06 ± 48.98

TyG, triglyceride–glucose index; FIB-4—Fibrosis-4 index; UACR, urinary albumin-to-creatinine ratio.

**Table 2:** Comparison of demographic and clinical variables between cardiometabolic patients (group 1) and patients with other comorbidities (group 2), all with psychiatric treatment.

Variable	Mean ± SD (Group 1) Cardiometabolic	Mean ± SD (Group 2) Other Comorbidities	Normality (Shapiro–Wilk)	Statistical Test	*p*-Value
Age (years)	63.21 ± 15.45	66.27 ± 13.70	Non-normal in both groups	Mann–Whitney	*p* = 0.310
Number of medications	5.83 ± 2.53	6.72 ± 2.36	Normal in both groups	t-test	*p* = 0.041
TyG index	8.96 ± 1.46	9.02 ± 1.10	Non-normal	Mann–Whitney	*p* = 0.880
FIB-4	3.62 ± 14.38	1.87 ± 5.60	Non-normal and highly skewed	Mann–Whitney	*p* = 0.650
Serum creatinine (mg/dL)	1.24 ± 2.82	1.10 ± 0.56	Non-normal	Mann–Whitney	*p* = 0.570
UACR (mg/g)	16.93 ± 24.79	33.51 ± 56.55	Non-normal	Mann–Whitney	*p* = 0.150

ANOVA, analysis of variance; CKD, chronic kidney disease; eGFR, estimated glomerular filtration rate; IQR, interquartile range; NCR-SI, FIB-4—Fibrosis-4 index, Neuro–Cardio–Renal Stress Index; SD, standard deviation; TyG, triglyceride–glucose index; UACR, urinary albumin-to-creatinine ratio.

**Table 3:** Comparison of demographic, renal, and metabolic parameters between the study groups.

Parameters	N	Group 1		Group 2		Kruskal–Wallis H	Asymp. Sig.
		Mean	SD	Mean	SD		
Systolic BP	143	152.77	21.14	141.33	17.18	7.005	0.008
Diastolic BP		95.21	16.86	86.04	10.64	10.224	0.001
Triglycerides		183.47	255.27	149.89	105.25	0.135	0.713
TyG index		9.13	0.79	9.09	0.74	0.001	0.979
ALT		26.34	13.52	25.73	14.84	0.086	0.770
AST		26.09	14.13	23.12	8.74	0.574	0.449
FIB-4		1.54	2.14	1.16	0.52	0.485	0.486
Serum Creatinine		0.83	0.19	1.06	0.38	10.408	0.001
eGFR		89.50	26.13	56.53	25.28	42.910	<0.001
UACR		17.32	24.69	33.53	56.54	1.815	0.178

ALT—Alanine aminotransferase, AST—Aspartate aminotransferase, BP—Blood pressure, eGFR—Estimated glomerular filtration rate, FIB-4—Fibrosis-4 index, SD—Standard deviation, TyG index—Triglyceride–glucose index (surrogate marker of insulin resistance), UACR—Urine albumin-to-creatinine ratio.

**Table 4:** Spearman correlation coefficients (ρ) among cardiometabolic, hepatic, and renal biomarkers in study groups.

Variable	TyG	FIB-4	Creatinine	UACR
TyG index	1.00	−0.41	0.02 **	0.17
FIB-4 score	−0.41	1.00	0.23	0.14
Creatinine (mg/dL)	0.02 **	0.23	1.00	−0.00 **
UACR (mg/g)	0.17	0.14	−0.00 **	1.00

Spearman’s ρ was used due to non-normal data distribution. Values reflect pairwise complete-case analysis. ** Correlation is significant at the 0.01 level (2-tailed).

## Data Availability

The data presented in this study are available on request from the corresponding author. The data are not publicly available due to privacy and ethical restrictions.

## Abbreviations

The following abbreviations are used in this manuscript:

NCR-SI	Neuro–Cardio–Renal Stress Index
N	Neuro-psychiatric domain score
C	Cardio-metabolic domain score
R	Renal domain score
TyG index	Triglyceride-Glucose Index
UACR	Urinary Albumin-to-Creatinine Ratio
eGFR	Estimated Glomerular Filtration Rate
AST	Aspartate Aminotransferase
ALT	Alanine Aminotransferase
BP	Blood Pressure
CKD	Chronic Kidney Disease
SD	Standard Deviation
IQR	Interquartile Range
KW test	Kruskal–Wallis Test

## References

[1] Chew N.W.S., Ng C.H., Tan D.J.H., Kong G., Lin C., Chin Y.H., Lim W.H., Huang D.Q., Quek J., Fu C.E. (2023). The global burden of metabolic disease: Data from 2000 to 2019. Cell Metab..

[2] Ye J., Xie Y., Gong Y. (2025). Association between cardiometabolic index and cardiometabolic multimorbidity in non-alcoholic fatty liver disease patients: Evidence from a cross-sectional study. Sci. Rep..

[3] Theodorakis N., Nikolaou M. (2025). From Cardiovascular-Kidney-Metabolic Syndrome to Cardiovascular-Renal-Hepatic-Metabolic Syndrome: Proposing an Expanded Framework. Biomolecules.

[4] Deste G., Lombardi C.M. (2023). Editorial: Cardiometabolic disease and psychiatric disorders. Front. Psychiatry.

[5] Pâslaru A.-M., Bounegru I., Laurențiu D., Ciubară A. (2025). Psychiatric Comorbidity, Functional Status, and Neuroinflammatory Pathways in Cancer Patients with and Without Type 2 Diabetes. Diseases.

[6] Casey D.E. (2005). Metabolic issues and cardiovascular disease in patients with psychiatric disorders. Am. J. Med..

[7] Opel N., Hanssen R., Steinmann L.A., Foerster J., Köhler-Forsberg O., Hahn M., Ferretti F., Palmer C., Penninx B.W.J.H., Gold S.M. (2025). Clinical management of major depressive disorder with comorbid obesity. Lancet Psychiatry.

[8] Wang Z., Qian H., Zhong S., Gu T., Xu M., Yang Q. (2023). The relationship between triglyceride-glucose index and albuminuria in United States adults. Front. Endocrinol..

[9] Popoviciu M.S., Moldovan A., Dorobantu F.R., Domocos P.C., Mariș L., Trifan D.F., Ghitea T.C., Manole F. (2025). Integrative Assessment of TyG Index, FIB-4, and eGFR as Composite Predictors of Metabolic Risk Clusters in Adults. Metabolites.

[10] Zhou H., Huang H., Zhao H., Pang N., Huang M., Ou C., Lao M. (2025). The albumin-to-creatinine ratio predicts and explores potential mediation of mortality in metabolic dysfunction-associated steatotic liver disease in U.S. adults: Evidence from NHANES 1999–2018. BMC Gastroenterol..

[11] Li H., Miao X., Li Y. (2023). The Triglyceride Glucose (TyG) Index as a Sensible Marker for Identifying Insulin Resistance and Predicting Diabetic Kidney Disease. Med. Sci. Monit..

[12] Liang F., Shan X., Chen X., Yang B. (2025). The association between triglyceride-glucose index and its combination with post-stroke depression: NHANES 2005–2018. BMC Psychiatry.

[13] Cui X.J., Xie B., Zhou Y.F., Yi X.Q. (2025). The association of the triglyceride-glucose index and its changes with 5-year all-cause mortality in patients with depression. Front. Psychiatry.

[14] Wan W., Yu Y. (2024). Association between the triglyceride glucose index and depression: A meta-analysis. Front. Psychiatry.

[15] Han M.T.T., Thumvijit T., Kranrod C., Tokonami S., Choocheep K., Kumsaiyai W., Wuttiin Y., Punturee K., Pornprasert S., Chiampanichayakul S. (2025). Serum miRNA and Metabolomic Signatures of Residential Radon Exposure in Chiang Mai, Thailand. Toxics.

[16] Kukreja S., Kalra G., Shah N., Shrivastava A. (2013). Polypharmacy in psychiatry: A review. Mens. Sana. Monogr..

[17] Jerjes W., Ramsay D., Stevenson H., Lalji K. (2024). Mental Health Polypharmacy in “Non-Coded” Primary Care Patients: The Effect of Deprescribing. J. Clin. Med..

[18] Abosi O., Lopes S., Schmitz S., Fiedorowicz J.G. (2018). Cardiometabolic effects of psychotropic medications. Horm. Mol. Biol. Clin. Investig..

[19] Marano G., Traversi G., Romagnoli E., Catalano V., Lotrionte M., Abbate A., Biondi-Zoccai G., Mazza M. (2011). Cardiologic side effects of psychotropic drugs. J. Geriatr. Cardiol..

[20] Dopierała M., Nitz N., Król O., Wasicka-Przewoźna K., Schwermer K., Pawlaczyk K. (2025). New and Emerging Biomarkers in Chronic Kidney Disease. Biomedicines.

[21] Nida S., Khan D.A., Khan M.Q.A., Pervez M.A., Saleem S., Chaudhry N. (2025). Comparative Analysis of Metabolic and Inflammatory Biomarker Profiles in Phenotypes of Metabolic Dysfunction-Associated Fatty Liver Disease. Age.

[22] Neagu O.M., Ghitea T., Marian E., Vlase L., Vlase A.M., Ciavoi G., Fehér P., Pallag A., Bácskay I., Nemes D. (2023). Formulation and Characterization of Mucoadhesive Polymeric Films Containing Extracts of Taraxaci Folium and Matricariae Flos. Molecules.

[23] Ma H., Guo L., Huang D., Wang L., Guo L., Geng Q., Zhang M. (2016). The Role of the Myocardial Microvasculature in Mental Stress-Induced Myocardial Ischemia. Clin. Cardiol..

[24] Nowroozpoor A., Gutterman D., Safdar B. (2020). Is microvascular dysfunction a systemic disorder with common biomarkers found in the heart, brain, and kidneys?—A scoping review. Microvasc. Res..

[25] Ferreira L., Fisberg R.M., Sarti F.M., Rogero M.M. (2024). Association between Inflammatory and Metabolic Biomarkers and Common Mental Disorders among Adults: 2015 Health Survey of São Paulo, SP, Brazil. Metabolites.

[26] Chourpiliadis C., Zeng Y., Lovik A., Wei D., Valdimarsdóttir U., Song H., Hammar N., Fang F. (2024). Metabolic profile and long-term risk of depression, anxiety, and stress-related disorders. JAMA Netw. Open.

[27] Bartz S.K., Caldas M.C., Tomsa A., Krishnamurthy R., Bacha F. (2015). Urine albumin-to-creatinine ratio: A marker of early endothelial dysfunction in youth. J. Clin. Endocrinol. Metab..

[28] Gupta S.K., Kitch D., Tierney C., Melbourne K., Ha B., McComsey G.A., AIDS Clinical Trials Group Study A5224s Team (2015). Markers of renal disease and function are associated with systemic inflammation in HIV infection. HIV Med..

[29] Jiang Y., Zhao B., Wang X., Tang B., Peng H., Luo Z., Shen Y., Wang Z., Jiang Z., Wang J. (2025). UKB-MDRMF: A multi-disease risk and multimorbidity framework based on UK biobank data. Nat. Commun..

[30] Beridze G., Dai L., Carrero J.J., Marengoni A., Vetrano D.L., Calderón-Larrañaga A. (2025). Associations between multimorbidity and kidney function decline in old age: A population-based cohort study. J. Am. Geriatr. Soc..

[31] Zhou M., Liu M., Xue C. (2025). Dissecting the Cellular Heterogeneity Underlying Liver Diseases Through the Integration of GWASs and Single-Cell RNA Sequencing. Biology.

[32] Potra Cicalău G.I., Marcu O.A., Ghitea T.C., Ciavoi G., Iurcov R.C., Beiusanu C., Trifan D.F., Vicaș L.G., Ganea M. (2024). Study of Periodontal Bacteria in Diabetic Wistar Rats: Assessing the Anti-Inflammatory Effects of Carvacrol and Magnolol Hydrogels. Biomedicines.

[33] Cesare M., D’ Agostino F., Damiani G., Nurchis M.C., Ricciardi W., Cocchieri A. (2025). Exploring the Impact of Medical Complexity on Nursing Complexity of Care in Paediatric Patients: A Retrospective Observational Study. J. Clin. Nurs..

